# The Utility of Invengenx® Bovine Patch for Right Ventricular Outflow Tract (RVOT) Reconstruction and Augmentation in the Surgical Management of Tetralogy of Fallot (TOF): A Contemporary Study and Review of the Literature

**DOI:** 10.7759/cureus.46882

**Published:** 2023-10-12

**Authors:** Vishal V Bhende, Tanishq S Sharma, Mathangi Krishnakumar, Anikode Subramanian Ramaswamy, Kanchan Bilgi, Sohilkhan R Pathan

**Affiliations:** 1 Pediatric Cardiac Surgery, Bhanubhai and Madhuben Patel Cardiac Centre, Shree Krishna Hospital, Bhaikaka University, Gokal Nagar, Karamsad, IND; 2 Community Medicine, SAL Institute of Medical Sciences, Ahmedabad, IND; 3 Anaesthesiology, St John's Medical College Hospital, Bengaluru, IND; 4 Pathology, PES Institute of Medical Sciences and Research, Kuppam, IND; 5 Neuroanaesthesiology, People Tree Hospitals, Bengaluru, IND; 6 Clinical Research Services, Bhanubhai and Madhuben Patel Cardiac Centre, Shree Krishna Hospital, Bhaikaka University, Gokal Nagar, Karamsad, IND

**Keywords:** invengenx® bovine patch, rvot reconstruction, tetralogy of fallot, pulmonary stenosis, cyanotic congenital heart disease

## Abstract

Background and objective

Complex congenital heart diseases (CHDs), such as the tetralogy of Fallot (TOF), often warrant reconstruction and augmentation of the right ventricular outflow tract (RVOT). This procedure requires the use of both synthetic and natural materials. However, finding the ideal material for tissue implants can be challenging. Biological materials often face issues such as tissue degeneration, calcium deposition, antigenicity, rejection, shrinkage, and fibrosis. These issues can lead to complications such as stenosis and insufficiency, potentially requiring early reoperations. In light of this, this study aimed to investigate the effectiveness of the Invengenx® bovine patch for RVOT reconstruction and augmentation.

Methods

This was a retrospective observational study conducted among eight children who underwent TOF correction cardiac surgery. Their demographic and clinical characteristics, intraoperative findings, and postoperative follow-up results at six months were collected from the hospital patient database.

Results

There were no deaths or complications in this study. We observed a significant reduction in the gradient across the pulmonary valve and the outflow tract at six months post-procedure. The analysis demonstrated that the Invengenx® bovine patch was successful and did not lead to any complications.

Conclusions

This study demonstrates the safety and efficacy of this engineered bovine pericardial patch (Invengenx®) as a cardiovascular substitute for surgical repair of both simple and more complex congenital cardiac defects.

## Introduction

The incidence of congenital heart diseases (CHDs) is not being reported accurately in India, and 200,000 children on average are born with them. Of these, over 20% require early surgical interventions [1]. Tetralogy of Fallot (TOF), which is one of the most common complex CHD, is characterized by four main features: pulmonary stenosis (PS), right ventricular hypertrophy, ventricular septal defect (VSD), and an overriding aorta. Contemporary surgical correction of TOF is usually done in the first year after birth and involves both closure of VSD and reconstruction of the right ventricular outflow tract (RVOT) [2]. The management of RVOT, which includes commissurotomy, patch expansion, complete reconstruction using a conduit, and infundibular myectomy, is contingent upon unique anatomical factors. Although valve-sparing surgery and transannular patch plasty are the frequently favored options, in some cases, reconstructive surgery involving a conduit may be required [2,3].

The repair and replacement of cardiovascular structures employ both synthetic and natural materials [4]. However, determining the optimal material for tissue implants remains a challenge. The use of biological materials often faces issues, such as tissue degeneration, calcium deposition, antigenicity, rejection, shrinkage, and fibrosis [5]. These often lead to stenosis and insufficiency, and may eventually result in early reoperation. Nevertheless, they hold potential as scaffolds that facilitate the growth of native tissue and support remodeling [5]. This advantage is particularly significant in comparison to synthetic materials when addressing RVOT reconstruction.

Invengenx® is a bovine pericardial tissue patch that is cross-linked by using a proprietary fixation methodology, elixP^TM^. This method preserves the natural collagen structures, thereby improving biological healing response with negligible bio-reactivity. elixP^TM^ preserves and strengthens the bonding of the helices within the individual collagen molecules and between other collagen molecules. This treatment leads to 100% cross-linking of the tissue and prevents unwanted effects, including suture line bleeding, delamination, and inflammatory response.

The present study aimed to assess the advantages and efficacy of a bovine patch (Invengenx®) for RVOT reconstruction and augmentation in the surgical management of TOF.

## Materials and methods

This study conducted at HM Patel Centre for Medical Care and Education, Anand, Gujarat, India, received approval from the Institutional Ethics Committee (IEC-2) vide IEC / BU / 2023 / Cr.35 /249/2023 dated 04.09.2023. The patients' parents provided written informed consent prior to the study for their data to be used. The study adhered to the guidelines outlined in the Declaration of Helsinki.

Objectives

The main objective was to assess the effectiveness of the Invengenx® bovine pericardial patch. Supplementary objectives encompassed evaluating the occurrence of stroke, infective endocarditis, renal failure, need for additional intervention, and mortality. The appraisal of implant performance required various diagnostic approaches, such as CT, echocardiography, MRI, Doppler ultrasonography, and angiography when deemed necessary.

Study population

Eight patients who underwent surgery for TOF correction with RVOT reconstruction using the Invengenx® patch by a single surgeon from 2022 to 2023 were included in the analyses. All patients received the standard of care as per the hospital policy. A transthoracic echocardiogram was performed at six months to identify patch-related complications, including surface thickening, surface leak, residual leak, vegetation, thrombus formation, calcification, aneurysmal dilatation, and residual shunt. Data on demographic and clinical characteristics, intraoperative findings, and postoperative follow-up results up to six months were collected from the hospital database.

Inclusion Criteria

The study included patients with cyanotic CHD, and grown-up CHD (TOF physiology) having RVOT obstruction including pulmonary atresia.

Exclusion Criteria

Patients with acyanotic CHD, requiring emergency cardiopulmonary bypass (CPB), were not included in the study.

Patch preparation

The Invengenx® bovine pericardial tissue patch is manufactured by Tisgenx Inc. located in California, USA, by a supremely focused team that ensures that the tissue patch needs are met.

**Figure 1:**
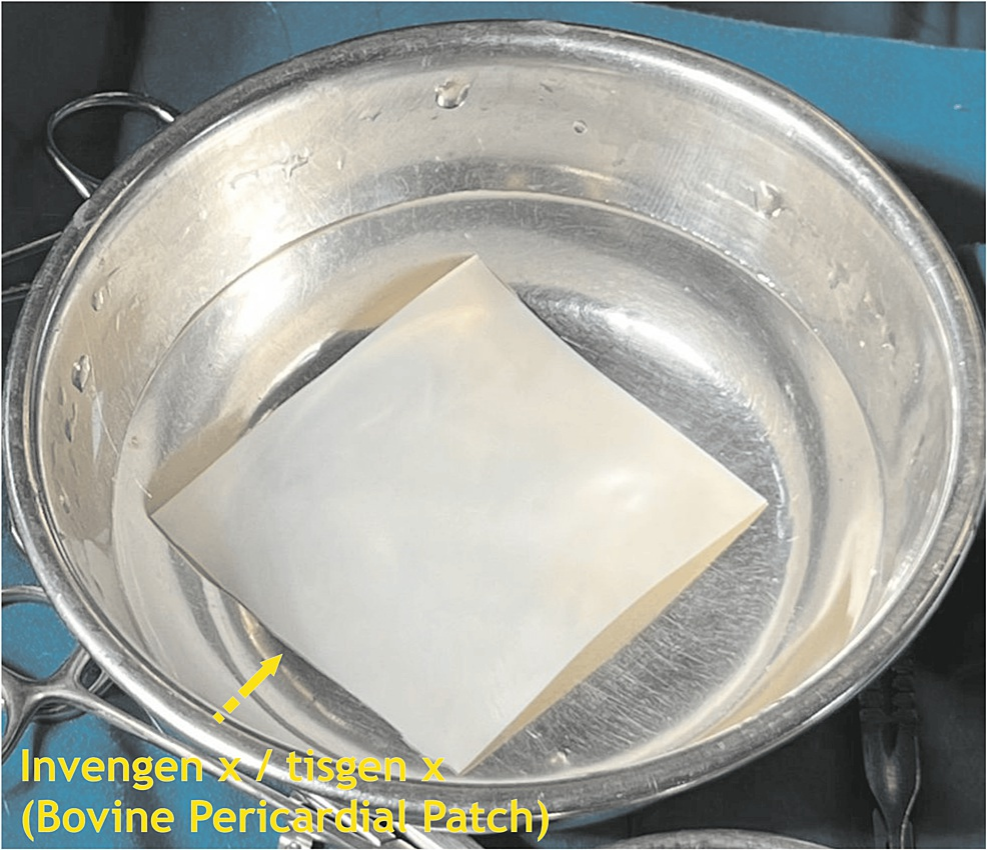
Invengenx® (Tisgenx) patch being prepared for implantation - image 1 Credits: Dr. Vishal V. Bhende

Invengenx® is fixed with the proprietary elixP^TM^ fixation technology. This preserves the triple helical structure of the individual collagen molecules (intermolecular) and between collagen fibrils (intramolecular). This fixation process achieves a complete reduction of antigenicity and preserves the tissue’s natural collagen formation. elixP^TM^ also enhances the tissue’s mechanical strength while maintaining its superior flexibility and soft and supple characteristics and prevents unwanted effects, such as suture line bleeding, delamination, and inflammatory response. Invengenx® bovine pericardial patch with a thickness of 0.5-0.75 mm and a size of 5 cm x 6 cm was used in all patients (Figure 1).

**Figure 2:**
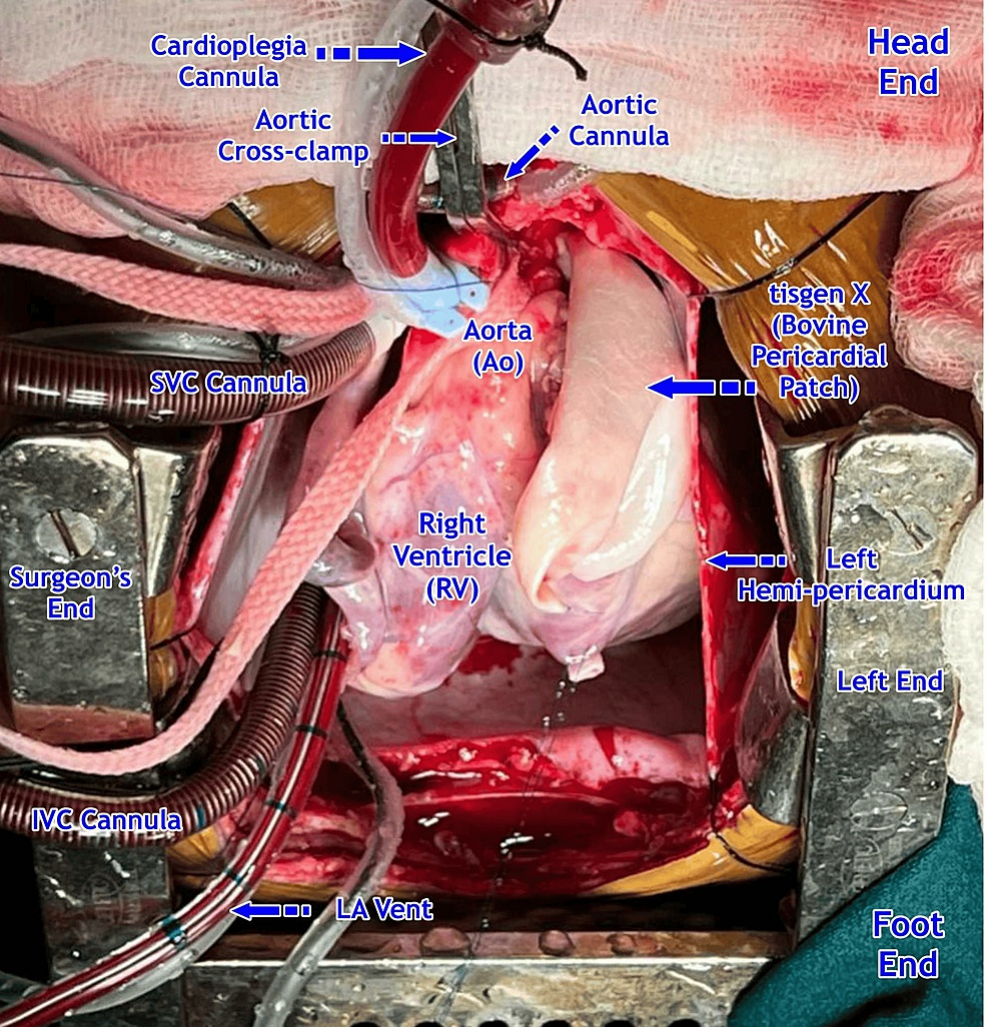
Invengenx® (Tisgenx) patch being prepared for implantation - image 2 SVC: superior vena cava; IVC: inferior vena cava; LA: left atrium Credits: Dr. Vishal V. Bhende

The placement in the RVOT is shown in Figure 2.

The features and properties of the patch are described in Video 1.

**Video 1:** Invengenx® (Tisgenx) patch demonstration Credits: Dr. Vishal V. Bhende

Statistical analysis

All data were analyzed after tabulation by using SPSS Statistics version 21 (IBM Corp., Armonk, NY). Quantitative data were presented as mean ± standard deviation or median (interquartile range), while qualitative data were presented as count or percentage, depending on the normality of data. The Wilcoxon rank-sum was conducted to compare the scenario pre and post-changes. A p-value <0.05 was considered statistically significant.

## Results

The study population consisted of eight patients: four males and four females. In all patients, the New York Heart Association classification before surgery was grade II. All patients underwent intracardiac Repair with RVOT reconstruction using a 0.1 mm polytetrafluoroethylene (PTFE) monocusp valve augmented with a transannular Invengenx® pericardial bovine patch. In addition, two patients underwent atrial septal defect (ASD) closure, three patients underwent patent ductus arteriosus (PDA) ligation, and a pulmonary valve-sparing technique was used in two patients. Details of all the patients are shown in Table 1.

**Table 1:** Masterchart of patients enrolled in the study P: patient; CPB: cardiopulmonary bypass; PVI: pulmonary vein index; CSICU: cardiac surgical intensive care unit; M: male; F: female; MAPCAs: major aortopulmonary collateral arteries; RVOT: right ventricular outflow tract; PTFE: polytetrafluoroethylene; PV annulus: pulmonary valve annulus; RPA: right pulmonary artery; LPA: left pulmonary artery; TOF: tetralogy of Fallot; VSD: ventricular septal defect; PS: pulmonary stenosis; PDA: patent ductus arteriosus: surgery 1: intracardiac repair with RVOT reconstruction using 0.1 mm PTFE monocusp valve augmented with transannular Invengenx® pericardial bovine patch

Patient ID	Age, years	Sex	Height, cm	Weight, kg	Diagnosis	Surgical procedure	Additional pathologies			Z score			CPB time, minutes	Cross-clamping time, minutes	Hypothermia, ^o^C	McGoon ratio	Nakata index, mm^2^/m^2^	PVI (mm^2^/m^2^)	RVOT gradients		Extubation, hours	Length of CSICU stay, days	Hospital stay, days
							MAPCAs	ASD	PDA	PV annulus	RPA	LPA							Preoperative	Postoperative			
P1	6	M	107	14.8	TOF + VSD + PS +MAPCAs	Surgery 1 + autologous treated pericardial patch closure of atrial septal defect	--	+	--	-2.71	+0.18	-2.38	205	164	24.5	1.7	298.4	228.9	69	16	193	12	15
P2	3	M	84	9	TOF + VSD + PS	Surgery 1 + autologous treated pericardial patch closure of atrial septal defect	--	+	--	-1.62	+0.64	+0.70	183	143	26	1.863	408.86	476.81	72	28	144	9	14
P3	8	F	111	14.450	TOF + VSD + PS + PDA + MAPCAs	Surgery 1 with pulmonary annular valve sparing + PDA ligation	+	--	+	-1.64	-0.55	+1.22	148	103	26	1.86	294.62	509.85	80	18	26	4	8
P4	4	F	84	10.5	TOF + VSD + PS; single left kidney	Surgery 1	--	--	--	-1.80	+0.73	+3.14	189	143	26	1.86	407.5	188.1	68	Nil	26	11	14
P5	4	M	97.5	11	TOF + VSD + PS + PDA	Surgery 1 + PDA ligation	--	--	+	-0.36	+0.05	+2.24	254	186	22	1.82	223.2	190.8	82	Nil	139	10	17
P6	3	F	82.5	7.7	TOF + VSD + PS + MAPCAs	Surgery 1 with pulmonary annular valve sparing	+	--	--	-0.52	+0.48	+2.17	169	119	28	1.82	317.6	521.2	82	Nil	143	10	14
P7	3	M	89	12.3	TOF + VSD + PS + anomalous left anterior descending coronary artery (LAD) crossing RVOT, MAPCAs	Surgery 1	+	--	--	-3.31	+0.54	+0.38	212	161	22	1.491	395.42	633.12	62	32	180	12	20
P8	3	F	85	10	TOF + VSD + pulmonary atresia, PDA+ MAPCAs, LAD coronary artery crossing RVOT	Rastelli operation with 14-mm hand-made PTFE goretex bi-cuspid valved conduit + PDA ligation	+	--	+	Atresia	+2.39	+0.93	191	247	26	1.41	323.6	510	Nil	68	150	10	15

The baseline and preoperative characteristics are shown in Table 2.

**Table 2:** Patient demographic characteristics IQR: interquartile range; TOF: tetralogy of Fallot; VSD: ventricular septal defect; PS: pulmonary stenosis; MAPCAs: major aortopulmonary collateral arteries; PDA: patent ductus arteriosus; n: number

Sr. no.	Description	Patient values
01	Age, years, median (IQR)	3.5 (3–4.5)
02	Sex, male/female, n	4/4
03	Weight, Kg, median (IQR)	11 (9.5–12.5)
	Diagnosis	
04	TOF + VSD + PS, n	8
	Additional pathology	
05	MAPCAs, n	3
06	PDA, n	2

**Figure 3:**
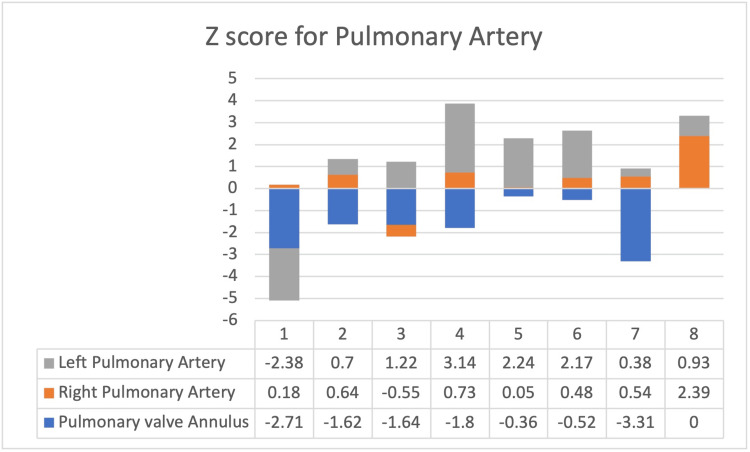
Echo z score of pulmonary artery profile The calculated z score is given as numbers Credits: Dr. Mathangi Krishnakumar

Altogether, eight patients underwent the procedure. The median age of the patients was 3.5 years. All patients had TOF-associated VSD and PS. The most common type of VSD was the perimembranous type (5/8). Preoperative echocardiogram showed a pulmonary gradient ranging from 62 to 82 mmHg. The z score of each patient is shown in Figure 3.

All patients had good biventricular function. The PS was valvular, supravalvular, and subvalvular. Two patients had infundibular stenosis.The intraoperative and postoperative features are summarized in Table 3. RVOT reconstruction was done using a 0.1 mm PTFE and the monocusp valve was augmented with an Invengenx® bovine pericardial patch in seven patients. One patient underwent a Rastelli procedure. The RVOT gradient measurements were available for four patients in both preoperative and postoperative periods. There was a significant reduction in the gradient in the postoperative period by a median of 68.9% (58-77), and this was statistically significant (p<0.001). The median cross-clamping time and CPB time were 152 and 190 minutes respectively with hypothermia maintained between 24 and 26 ^o^C. The McGoon ratio, Nakata index, and pulmonary vein index (PVI) are presented in Table 3.

**Table 3:** Intraoperative and postoperative characteristics IQR: interquartile range; CPB: cardiopulmonary bypass; PVI: pulmonary vein index; ICU: intensive care unit

Sr. no.	Description	Patient values, median (IQR)
01	Cross-clamping time, minutes	152 (131–169)
02	CPB time, minutes	190 (176–206)
03	Hypothermia, °C	26 (24–26)
04	McGoon ratio	1.82 (1.6–1.86)
05	Nakata index, mm^2^/m^2^	320.6 (306–398)
06	PVI, mm^2^/m^2^	493 (333–511)
07	Extubation, days	6.5 (5.5–12.5)
08	ICU stay, days	10 (9.5–11.25)
09	Hospital stay, days	14.5 (14–15.5)

There were no documented intraoperative complications. The patch preparation and handling were rated as good by the surgeon in all cases. All patients underwent extubation in the ICU with a mean duration of 6.5 days. The duration of ICU and hospital stay are presented in Table 3. There were no perioperative complications, such as stroke, infective endocarditis, renal failure, or mortality. There was a significant reduction in the gradient across the pulmonary valve and the outflow tract at six months post-procedure. These changes were statistically significant (p<0.001). The median pulmonary gradient was 18.5 (18-20) mmHg at six months. There were no patch-related complications, such as surface thickening, surface leak, residual leak, vegetation, thrombus formation, calcification, aneurysmal dilatation, or residual shunt at six months post-procedure.

## Discussion

Our findings revealed that the Invengenx® pericardial bovine patch is a viable option for RVOT reconstruction and augmentation in the surgical management of TOF. At the six-month follow-up, no patch-related complications were observed. RVOT augmentation using various synthetic and non-synthetic materials has been described in previous studies. Each material has its own advantages and disadvantages. The available data are largely skewed by the retrospective, single-centered nature of the studies and the fact that these studies have a significant bias based on the surgeon’s preference of material [5]. Historically, materials, including PTFE, commonly referred to as Teflon, and expanded polytetrafluoroethylene (ePTFE), sold under the brand name Gore-Tex, have been widely utilized for patches in the context of RVOT and pulmonary artery. These microporous materials are notable for their relatively positive biocompatibility characteristics and are linked to diminished fibrosis when compared to earlier synthetic counterparts, such as polyvinyl (Ivalon) [6].

Over time, the autologous pericardium was reported to be superior to Ivalon and to be at par with PTFE [6]. It had the advantage of being cost-effective and easily available during surgery and having the least risk for rejection. However, in TOF cases, where there is considerable probability of reoperation, the availability becomes a major hindrance. The amount of tissue available with regard to sequential surgical interventions can be problematic and there is a risk of aneurysm formation [7]. Tissue processing with glutaraldehyde has been shown to reduce the incidence of aneurysmal dilatation [8]. Homograft pericardium is considered less efficient than autologous pericardium in terms of graft performance; however, its greater availability makes it a sought-after option. The handling characteristics of both materials are similar, and the main complication encountered is calcification. This is mainly due to the antigenicity causing an increased inflammatory response, leading to calcium deposition [9].

Bovine pericardium, in comparison, demonstrates lower antigenicity and offers better surgical characteristics. It is smooth and easy to handle thanks to reduced elasticity and favorable stiffness. Bovine pericardium, when left untreated, can degrade partially and become inflamed. To improve the mechanical properties and reduce inflammation, various processing methods have been explored. These methods mainly focus on changing the cross-linking [10]. Various studies have shown that photooxidation methods and pretreatment with glutaraldehyde decrease degradation. The major disadvantage of the fixation method is that it increases the calcium deposition rate. The mechanism is thought to be due to the remnant tissue antigenicity post-processing, which triggers an inflammatory response. Recently, an anti-calcification tissue engineering process (ADAPT) was shown to be an effective alternative [11].

Several studies have utilized bovine pericardial patches for RVOT reconstruction, showing varying rates of complication [12-19]. Abdulali et al. demonstrated the potential safety of bovine monocusp patches [12]. The bovine jugular vein graft used for RVOT reconstruction was found to have complications of valve thrombosis. The associated mortality ranged from 5 to 10% [14-15]. The Contegra valved conduits showed improved mortality rates in both mid and long-term follow-ups. The complication reported with this device was the occurrence of stenosis [16-19]. However, the complication rates were lower in comparison with cryopreserved aortic homografts [20]. PTFE-constructed conduits have been associated with increased occurrence of reoperation due to bleeding at the point of graft and stenosis [6,21]. More recent studies have shown the superiority of bovine patches with modified preparation techniques and technology. The complication rates and mortality are significantly lower when compared to its use two decades back [22,23].

The Invengenx® bovine pericardial patch uses a special fixation method called elixP^TM^, which preserves and strengthens the bonding of the helices within the individual collagen molecules and between other collagen molecules. This advanced treatment leads to superior biocompatibility with the host tissue and prevents degradation. The findings of our study contribute to the sparse literature on materials used for RVOT reconstruction. It provides valuable insights into the utility of the Invengenx® bovine pericardial patch as an appropriate alternative with many advantages in patients with TOF undergoing RVOT reconstruction. The special features of the patch are summarized in Table 4.

**Table 4:** Salient features of Invengenx® pericardial bovine patch

Sr. no.	Description
01	Uniform collagen thickness over the entire patch
02	Superior biocompatibility
03	Exceptional tensile and suture retention strength
04	Does not need special sutures
05	Intact matrix membrane
06	Minimal rinsing time
07	Conforms to vasculature
08	Easy to handle
09	Cost-effective
10	Resists delamination
11	Extremely elastic and pliable

Various studies published over the past three decades that demonstrate the use of bovine patches for RVOT reconstruction and associated complications are shown in Table 5.

**Table 5:** Studies on various bovine patches used for RVOT reconstruction and complications BPMP: bovine pericardial monocusp patch; CBJ: Contegra® bovine jugular vein graft; CVC: Contegra® valved conduits; HG: aortic homografts; PTFE: polytetrafluoroethylene

Author	Year	Patch used	Number of patients	Complications	Mortality
Abdulali et al. [12]	1985	BPMP	21	Nil	3
Gundry et al. [13]	1994	BPMP	19	Nil	1
Chatzis et al. [14]	2003	CBJ	15	1 (valve thrombosis)	0
Dave et al. [15]	2005	CBJ	93	12 (11 stenosis, 1 somatic growth)	5
Göber et al. [16]	2005	CVC	38	5 (stenosis)	1
Sierra et al. [17]	2007	CVC	50	Nil	
Breymann et al. [18]	2009	CVC	165	Nil	0
Christenson et al. [20]	2010	HG	120	9 (stenosis)	1
Sfyridis et al. [19]	2011	CVC	34	1 (thrombosis)	0
Talwar et al. [6]	2017	PTFE	50	6 (bleeding)	1
Qian et al. [21]	2022	PTFE-valved	21	2 (Stenosis)	Nil
Huang et al. [22]	2023	Single-valved bovine pericardium patch (svBPP)	88	1 (infective endocarditis)	1
Di Pasquale et al. [23]	2023	Contegra monocusp together with delamination of native leaflet tissue	18	Nil	Nil

Limitations

This study has a few limitations, primarily related to its single-center, retrospective design and the fact that it involved a small, select group of patients. The Invengenx® pericardial bovine patch was used only for RVOT reconstruction. Hence, multi-centric studies with different pathologies and larger sample sizes have to be conducted to apply the findings of this study for other indications. Since this was not a comparative study, it did not employ the use of a different material. Further studies may be undertaken to understand the differences between the outcomes with different varieties of patch materials.

## Conclusions

Many options are available for surgical procedures in children born with CHD. However, the materials used in the surgery can degrade over time, which often leads to the need for repeat surgical interventions. This study involved a preliminary analysis of the use of Invengenx® bovine pericardial patch for RVOT augmentation in the surgical management of TOF. Our results indicate that this technique can be a durable and affordable option in resource-limited settings while maintaining good outcomes and enhancing patient safety. However, further studies with a longer follow-up period are necessary to validate our findings.
